# An 11-Year-Old Athlete With Dynamic Stenosis of Coronary Artery Anomaly With Normal FFR-Dobutamine But Abnormal iFR-Dobutamine

**DOI:** 10.1016/j.jaccas.2026.107028

**Published:** 2026-03-25

**Authors:** Anselm W. Stark, Ali Mokhtari, Ryota Kakizaki, Marius R. Bigler, Matthias Siepe, Isaac Shiri, Lorenz Räber, Christoph Gräni

**Affiliations:** a Department of Cardiology, Inselspital, Bern University Hospital, University of Bern, Bern, Switzerland; b Department of Cardiac Surgery, Inselspital, Bern University Hospital, University of Bern, Bern, Switzerland

**Keywords:** AAOCA, ACAOS, anatomical high-risk features, anomalous coronary artery, AORCA, CCTA, coronary artery anomaly, dobutamine, FFR, iFR, interarterial, intramural, IVUS, R-AAOCA, R-ACAOS

## Abstract

**Background:**

Anomalous aortic origin of a right coronary artery (R-AAOCA) can cause stress-induced ischemia owing to dynamic stenosis of the intramural course. While fractional flow reserve during dobutamine challenge (FFR_Dobutamine_) is the reference standard for hemodynamic assessment, specific scenarios favoring instantaneous wave-free ratio (iFR) over FFR remain unclear.

**Case Summary:**

An 11-year-old male competitive basketball athlete with exertional angina and R-AAOCA underwent invasive angiography. Intravascular ultrasound showed a reduction in the intramural coronary lumen cross-sectional areafrom rest to dobutamine stress (from 4.6 to 3.1 mm^2^ in systole; from 7.6 to 3.7 mm^2^ in diastole), while FFR_Dobutamine_ was 0.85 and iFR_Dobutamine_ was 0.72.

**Discussion:**

The patient's iFR_Dobutamine_ matched geometrical changes and clinical presentation, while his FFR_Dobutamine_ underestimated ischemia.

**Take-Home Message:**

In the case of predominant diastolic, stress-induced intramural deformation of the R-AAOCA vessel, iFR_Dobutamine_—targeting diastolic stress-pressure ratio—may be preferred over FFR_Dobutamine_, as the latter may miss ischemia given its cardiac cycle–averaged hyperemic calculations.

## Central Illustration

**Figure:**
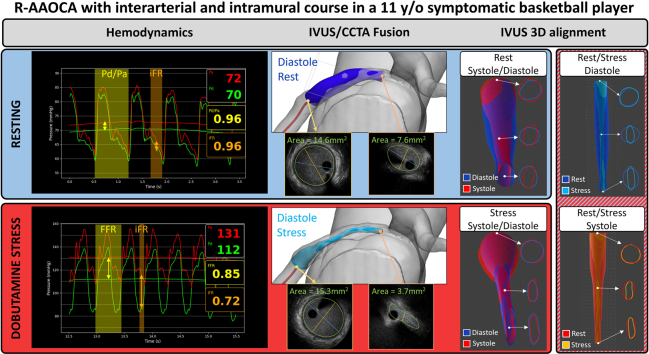

**Visual Summary:**
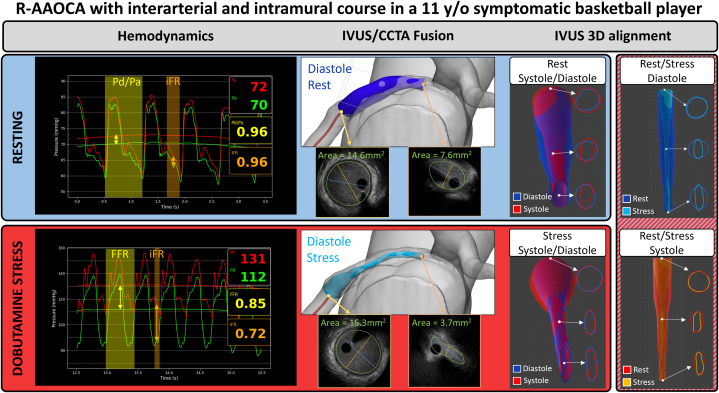
R-AAOCA With Interarterial and Intramural Course in an 11-Year-Old Symptomatic Basketball Player (Top Left, blue background) Patient-specific invasive findings at rest demonstrate a Pd/Pa ratio of 0.96 and an iFR of 0.96. Diastolic IVUS reveals a vessel with a minimal lumen area of 6.6 mm^2^ during diastole (distal reference: 14.6 mm^2^), with the 3D reconstruction coregistered to the CCTA. The vessel displays significant geometrical changes between diastole and systole. (Bottom Left, red background) Under dobutamine stress, invasive findings show a hemodynamically nonsignificant FFR_Dobutamine_ of 0.85, contrasted by a significantly lower iFR_Dobutamine_ of 0.72. IVUS shows a marked reduction in lumen area to 3.7 mm^2^ (distal reference: 15.3 mm^2^). (Right Side, red/blue striped background) Comparative analysis from rest to stress indicates that luminal deformation is significantly more pronounced during the diastolic phase than the systolic phase. This phase-specific dynamic likely explains the discrepancy between the cycle-averaged FFR_Dobutamine_ and the diastolic-specific iFR_Dobutamine_. 3D = three-dimensional; CCTA = coronary computed tomography angiography; FFR = fractional flow reserve; iFR = instantaneous wave-free ratio; IVUS = intravascular ultrasound; MLA = minimal lumen area; Pd/Pa = distal coronary artery pressure/aortic pressure; R-AAOCA = anomalous aortic origin of a right coronary artery.

## History of Presentation

An 11-year-old male competitive basketball player (team training about 5 times per week and monthly competitions) presented to our coronary artery anomaly clinic for evaluation of an anomalous aortic origin of a right coronary artery (R-AAOCA). At our clinics, he underwent a full work-up including low-dose rubidium positron emission tomography (Rb-PET) during dobutamine and invasive angiography with dobutamine-atropine volume challenge, intravascular ultrasound (IVUS), and intracoronary pressure measurements to determine hemodynamic relevance and guide management.

## Past Medical History

**Figure 1:**
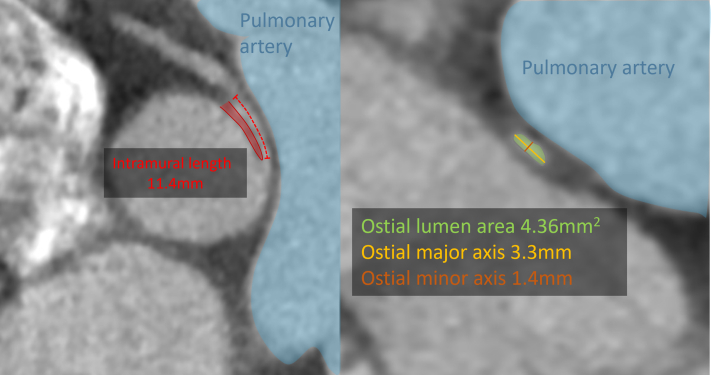
CCTA Images (Left) Intramural course length of 11.4 mm, with the interluminal space between the aorta and the coronary artery highlighted in red and the pulmonary artery in blue. (Right) Ostial lumen area of 4.36 mm^2^, with a major axis of 3.3 mm and a minor axis of 1.4 mm. The pulmonary artery is again shown in blue. CCTA = coronary computed tomography angiography.

The patient first came to clinical attention at 2.5 years of age, when R-AAOCA was identified by echocardiography during evaluation for suspected Kawasaki disease, leading to conservative management. At that time, and on subsequent follow-up, no coronary artery aneurysms, dilatation, or other Kawasaki-related coronary changes were identified. With the onset of basketball training, he developed recurrent exertional chest pain at age 9. Coronary computed tomography angiography (CCTA) confirmed R-AAOCA with anatomic high-risk features: an acute take-off angle of 34°, a small ostial area of 4.36 mm^2^, an elliptical ostium (major/minor 3.3/1.4 mm; ratio: 2.4), an intramural segment measuring 11.4 mm in length, and a distal reference lumen area of 12 mm^2^ (corresponding to ∼60% stenosis) (Figure 1). Bicycle ergometry did not show clear evidence of ischemia on electrocardiogram; however, the patient continued to experience exertional angina during intensive physical activity over the subsequent 2 years, with symptoms most recently during a basketball training camp several weeks before referral. He had no cardiovascular risk factors, no prior surgery, and was not taking any regular medications.

## Differential Diagnosis

The leading diagnosis was symptomatic R-AAOCA with an intramural course causing exertional ischemia. Because symptoms in R-AAOCA are often unreliable^1^ and adverse events may occur without prior warning, alternative causes of exertional chest pain were briefly considered. Exercise-induced arrhythmia, bronchospasm, and musculoskeletal pain were deemed unlikely given noninvasive and invasive evaluations and reproducible ischemic symptoms. Myocarditis was excluded based on absence of myocardial scar, as well as normal biomarkers, electrocardiogram, and wall-motion analysis.

## Investigations

### Noninvasive imaging

**Figure 2:**
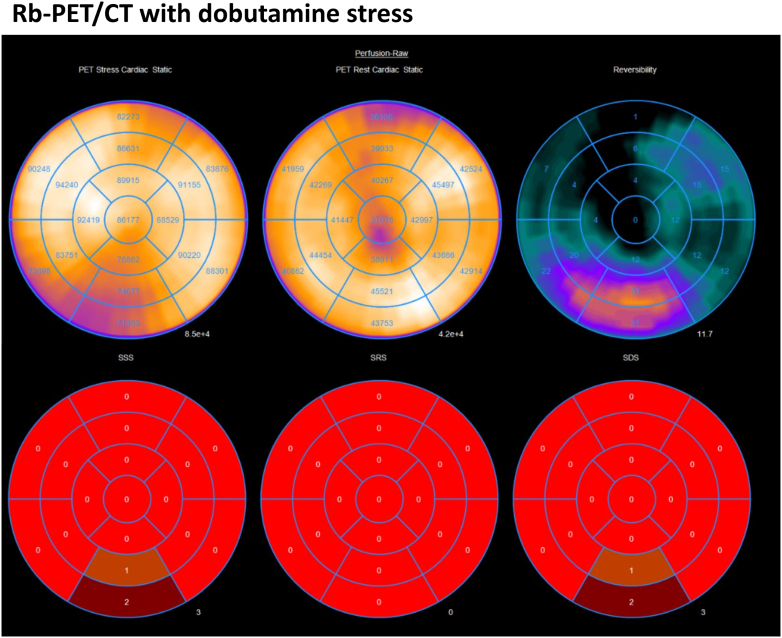
Rb-PET/CT During Dobutamine Stress (Left) Rb-PET/CT stress perfusion map during dobutamine-atropine volume challenge showing a reversible perfusion defect in the inferior myocardium (AHA segments 4 and 9), corresponding to ∼5% of left ventricular mass. (Right) Matched rest perfusion demonstrating full reversibility. Images are shown in short-axis orientation with the same intensity scale. AHA = American Heart Association; Rb-PET/CT = rubidium positron emission tomography/computed tomography.

A low-dose Rb-PET was performed with dobutamine stress. Maximal heart rate of 181 beats/min (87% of predicted) was achieved with maximal blood pressure of 123/60 mm Hg. There was a small perfusion deficit (5% of left ventricular myocardium) within the distal perfusion territory of a right-dominant R-AAOCA, without any scar tissue (Figure 2).

### Invasive assessment

#### Resting conditions

Invasive coronary angiography was performed via radial access using a JL 6.5-F sheathless catheter, without additional sedation. At rest, IVUS (Video 1) demonstrated a minimal lumen area (MLA) of 4.6 mm^2^ (heart rate: 88 beats/min, blood pressure: 90/60 mm Hg). The instantaneous wave-free ratio (iFR) is a vasodilator-free index of coronary lesion severity, calculated as the ratio of distal coronary artery pressure to aortic pressure (Pd/Pa) during the diastolic “wave-free” period, when microvascular resistance is minimal. Resting Pd/Pa and iFR were both normal at 0.96.

#### Adenosine stress

**Figure 3:**
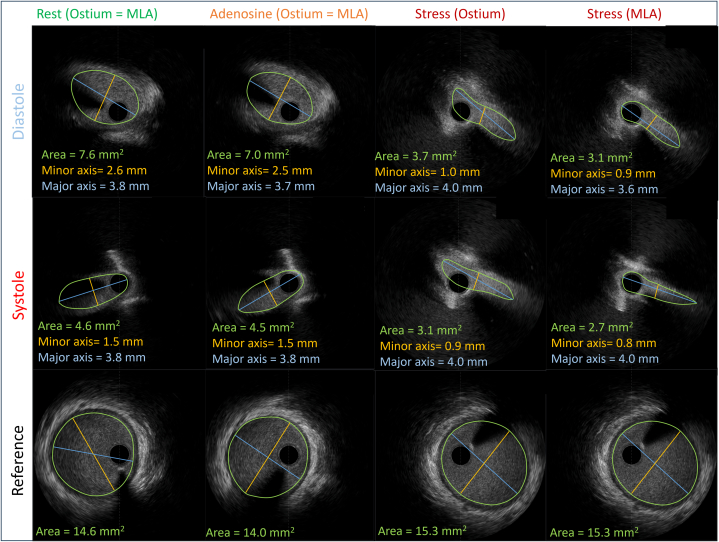
IVUS Ostial Cross-Sections (Diastole and Systole) at Rest, During Adenosine, and During Dobutamine Stress (First Column) At rest: diastolic lumen area 7.6 mm^2^ and systolic lumen area 4.6 mm^2^ (reference area: 14.6 mm^2^). (Second Column) Adenosine: diastolic 7.0 mm^2^ and systolic 4.5 mm^2^ (reference area: 14.0 mm^2^). (Third Column) Dobutamine volume challenge (stress ostium): diastolic 3.7 mm^2^ and systolic 3.1 mm^2^. (Fourth Column) Dobutamine volume challenge (stress ostium): diastolic 3.1 mm^2^ and systolic 2.7 mm^2^. Note the marked reduction in diastolic lumen area during stress compared with at rest (7.6 → 3.7 mm^2^). Panels are aligned to the same scale. Lumen contours are in green, minor axis is in yellow, and the major axis is in blue. IVUS = intravascular ultrasound; MLA = minimal lumen area.

After intravenous adenosine infusion (140 μg/kg/min), the patient's blood pressure fell to 74/43 mm Hg and heart rate rose to 107 beats/min; the MLA remained ∼4.5 mm^2^, FFR during adenosine infusion (FFR_Adenosine_) was 0.94, and iFR_Adenosine_ was 0.89 (Figure 3, Table 1).

**Table 1:** Invasive Measurements

	Resting Conditions	Adenosine Conditions	Stress Conditions
Heart rate (beats/min)	88	107	171
Blood pressure (mm Hg)	90/60	74/43	154/100
Pd/Pa, FFR_Adenosine_, and FFR_Dobutamine_, respectively	0.96	0.94	0.85
iFR, iFR_Adenosine_, and iFR_Dobutamine_, respectively	0.96	0.89	0.72
IVUS MLA diastole (mm^2^)	7.4	7.0	3.7
IVUS MLA circumference diastole (mm)	10.0	9.7	9.1
IVUS MLA diameter stenosis diastole (%)	30	32	51
IVUS MLA elliptic ratio (major/minor) diastole	1.5	1.5	4.1
IVUS MLA minor axis length diastole (mm)	2.6	2.5	1.0
IVUS MLA major axis length diastole (mm)	3.8	3.7	3.9
IVUS MLA systole (mm^2^)	4.6	4.5	2.7
IVUS MLA circumference systole (mm)	8.8	8.7	8.7
IVUS MLA diameter stenosis systole (%)	45	45	62
IVUS MLA elliptic ratio (major/minor) systole	2.5	2.5	5.4
IVUS MLA minor axis length systole (mm)	1.5	1.5	0.8
IVUS MLA major axis length systole (mm)	3.8	3.8	4.0
Reference area (mm^2^)	14.6	14.0	15.3
Reference circumference (mm)	13.6	13.3	13.9
IVUS intramural length (mm) (estimated by elliptic ratio >1.3)	12	12	17

FFR = fractional flow reserve; iFR = instantaneous wave-free ratio; IVUS = intravascular ultrasound; MLA = minimal lumen area; Pd/Pa = distal coronary artery pressure/aortic pressure.

#### Dobutamine stress

**Figure 4:**
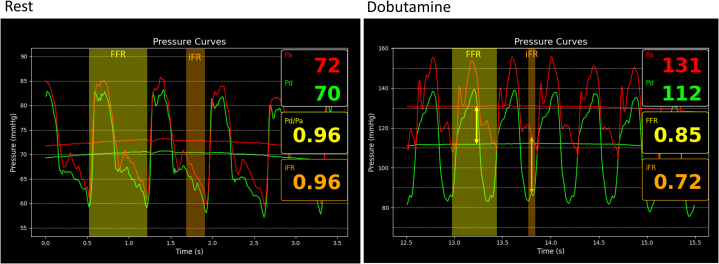
Pd/Pa, FFR, and iFR During Rest and Adenosine-Dobutamine Stress (Left) Pd/Pa and iFR (range displayed by orange bars in all images, while Pd/Pa or FFR is in yellow) during resting conditions, showing no discrepancies (both 0.96). (Right) During dobutamine stress there was a clear difference, with FFR of 0.85 and iFR of 0.72. FFR = fractional flow reserve; iFR = instantaneous wave-free ratio; MLA = minimal lumen area; Pd/Pa = distal coronary artery pressure/aortic pressure.

To reproduce exercise physiology, a dobutamine-atropine volume challenge was performed (dobutamine: 40 μg/kg/min, atropine: 1 mg, plus 3 L saline). Peak heart rate reached 171 beats/min, and blood pressure increased to 154/100 mm Hg. The angina was reproduced during the dobutamine challenge, with the same description of intense angina radiating to the teeth. Throughout the protocol, FFR_Dobutamine_ and iFR_Dobutamine_ were continuously assessed to evaluate hemodynamic relevance. Cycle-averaged FFR_Dobutamine_ decreased to 0.85; iFR_Dobutamine_ decreased to 0.72 (Figure 4). IVUS during peak dobutamine (Video 2) showed an MLA of 2.7 mm^2^ (reference area: 15.3 mm^2^). Effective percentage diameter stenosis was ∼62%, approximating a circular stenosis from the measured area (area *A* = 2.7 mm^2^ → diameter *D* ≈ 1.85 mm) and using the reference diameter (4.67 mm), with the formula (1 − [*D*_Stenosis_/*D*_Normal_] × 100) (Table 1).

### 3D reconstruction and IVUS/CCTA fusion

**Figure 5:**
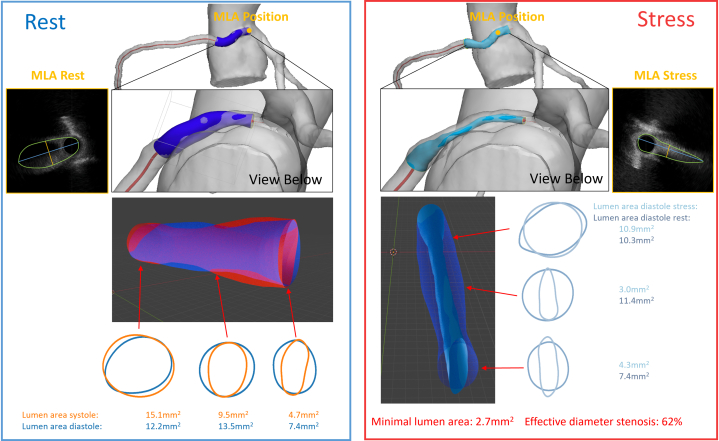
3-Dimensional Reconstruction of IVUS Images and Alignment With CCTA Images (Left) Diastolic IVUS reconstruction registered to the CCTA-derived centerline, showing the intramural course. (Bottom Inset) Difference map (diastole minus systole) demonstrating pulsatile lumen deformation along the intramural segment. (Right) Diastolic reconstruction during dobutamine stress showing marked lumen reduction throughout the intramural course (example midpoint area: 11.4 mm^2^ at rest → 3.0 mm^2^ during stress). CCTA = coronary computed tomography angiography; IVUS = intravascular ultrasound; MLA = minimal lumen area.

**Figure 6:**
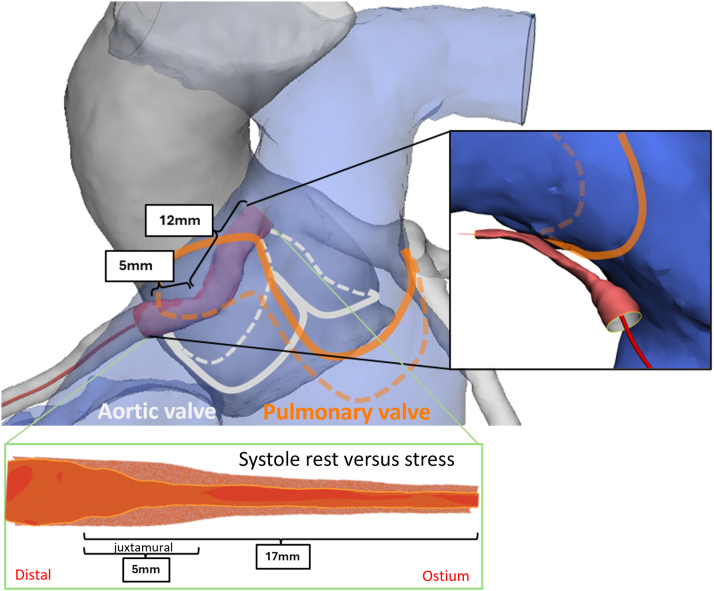
Anatomical Relation of the Anomalous Right Coronary Artery to the Pulmonary Valve and Aortic Commissures Systolic IVUS-based geometry (reconstructed during dobutamine stress) aligned to CCTA anatomy, illustrating the vessel course relative to the pulmonary (in orange) and aortic valve planes (in gray). A focal 5-mm segment that runs posterior to the pulmonary valve could account for the observed 5 mm increase in apparent intramural length during stress versus rest. CCTA = coronary computed tomography angiography; IVUS = Intravascular ultrasound.

Rest and stress pullbacks were segmented and gated (identifying systole/diastole) with the help of AIVUS-CAA software (version 1.0.0).^2^ The contour data were then reconstructed with the *multimodars* package (version 0.0.6),^3^ aligning systolic and diastolic frames during rest and stress^4^ and registering them with a CCTA-derived centerline (Figures 5 and 6). The 3D-reconstructed images during rest showed significant pulsatile lumen deformation (change from diastole to systole), where the vessel appeared almost circular over the full intramural length during diastole, then elliptic over a length of 12 mm during systole. The stress-induced lumen deformation was especially visible in diastole, with marked changes in the intramural cross-section from 11.4 mm^2^ at rest to 3.0 mm^2^ during stress (see Figure 5, Video 3, Video 4, Video 5, Video 6). Of note, at stress the intramural segment also appeared longer (17 mm) than measured at rest (12 mm). Careful centerline alignment and 3D reconstruction demonstrated that this 5-mm discrepancy corresponded to a short juxtamural segment (ie, the segment immediately adjacent to the aortic wall after exiting the intramural course) distal to a small kink in the right coronary artery that ran behind the level of the pulmonary valve. Together, these findings indicated a combination of intramural and an additional interarterial compression during stress. Multimodality imaging fusion helped reveal this dynamic behavior and helped with surgical planning (Figure 6).

## Management

After heart team discussion, considering the symptoms, competitive sports ambitions, the small area of ischemia on Rb-PET, and the low iFR_Dobutamine_ combined with stress-induced geometric lumen deformation, surgery was performed—despite a negative FFR_Dobutamine_ result, which remains the current gold standard. After surgical reimplantation of the R-AAOCA, the patient showed an uncomplicated postoperative course.

## Discussion

In R-AAOCA, intramural dynamic stenosis is the primary mechanism of stress-induced ischemia.^5^ FFR_Dobutamine_, which measures the cycle-averaged pressure gradient during dobutamine stress, is often considered the gold standard for hemodynamic assessment in this context.^6^ However, its performance may be suboptimal in lesions where the compression is mainly in diastole.

We present a case demonstrating a discrepancy between FFR_Dobutamine_ and iFR_Dobutamine_ in a symptomatic R-AAOCA. While FFR_Dobutamine_ was hemodynamically nonrelevant at 0.85, iFR_Dobutamine_ demonstrated hemodynamic relevance at 0.72 when applying the conventional FFR threshold of ≤0.8 to both indices. This discrepancy stems from the fundamental pathophysiology of coronary flow and the specific mechanics of the intramural segment. Coronary perfusion occurs predominantly during diastole, when the microvascular circulation is maximally perfused and intramyocardial resistance is lowest. The iFR specifically assesses the pressure gradient during diastole, and it is an established marker in fixed stenoses such as atherosclerosis. However, given that dynamic stenosis in AAOCA with an intramural course typically manifests under stress conditions, iFR assessed during dobutamine-induced stress may represent the preferred physiological parameter. Although in this athlete, IVUS revealed systolic compression, the marked stress-induced lumen deformation was in diastole. Under dobutamine stress, the diastolic lumen area was reduced by >50%, which created a critical stenosis specifically during the cardiac phase, where myocardial perfusion predominantly occurs. Cycle-averaged FFR_Dobutamine_ may “average out” this diastolic impairment with a less impaired systolic gradient, thereby underestimating the true hemodynamic impact. In contrast, iFR_Dobutamine_, by isolating the diastolic window, directly interrogates the vessel's function during the most vulnerable period, making it a more pathophysiologically aligned metric in this scenario. The concordance of the ischemic iFR_Dobutamine_ value with the patient's reproducible symptoms and the perfusion deficit on Rb-PET further supports this rationale.

While discrepancies between FFR_Adenosine_ and FFR_Dobutamine_ have been described,^7^ the comparison between iFR and FFR under dobutamine stress is novel in AAOCA. In the context of myocardial bridges, also displaying dynamic stenosis, iFR has already been proposed as a potentially superior hemodynamic marker^8^; however, no comparable analysis has been performed in AAOCA to date. Although no definitive threshold for iFR_Dobutamine_ has been established, and the commonly used cutoff of ≤0.89 cannot be directly applied to determine ischemia in AAOCA, the marked reduction in iFR observed during dobutamine infusion in this case provides supportive evidence that the AAOCA was responsible for the patient's exertional chest pain. This raises the key question of how to manage a symptomatic patient with high-risk anatomy and nonischemic FFR_Dobutamine_ but clear clinical and perfusion evidence of ischemia. Future studies should compare these indices with outcomes to determine which parameter best guides AAOCA management.

This case highlights the limitations of single-imaging modalities. Although CCTA identified high-risk anatomy, it could not quantify dynamic changes. Standard IVUS showed cross-sections but lacked full 3D and temporal context. PET helped to identify a perfusion deficit on a myocardial level^9^ but lacked information on vessel-based geometrical change and anatomy-based hemodynamic relevance. Advanced multimodality fusion was necessary to fully appreciate the vessel's complex course and its dynamic interaction with adjacent structures—such as the apparent stress-induced lengthening of the intramural segment due to a juxtamural kink behind the pulmonary valve. Tools such as the *multimodars* package^3^ may aid understanding, moving beyond area measurements to dynamic 4D assessment.

## Conclusions

The findings of this report indicate that iFR_Dobutamine_ may better detect diastolic, stress-induced ischemia in R-AAOCA than FFR_Dobutamine_.

**Take-Home Messages**

• In AAOCA with marked vessel dynamics, iFR_Dobutamine_ may better reflect hemodynamic significance than cycle-averaged FFR_Dobutamine_.
• Integrating multimodality imaging (IVUS, CCTA fusion, perfusion PET) with phase-sensitive pressure measurements improves diagnostic confidence and guides individualized management.

## Funding Support and Author Disclosures

Dr Kakizaki has received consulting fees from 10.13039/100018503Infraredx USA, speaker fees from Abbott Medical Japan, Boston Scientific Japan, Philips Japan, and 10.13039/501100020316Orbusneich Medical, and manuscript writing fees from Orbusneich Medical and Philips Japan, outside the submitted work. Dr Bigler has received grant support from the Bangerter-Rhyner-Foundation. Dr Räber has received funding to his institution from 10.13039/100000046Abbott, 10.13039/100008497Boston Scientific, 10.13039/501100005035Biotronik, 10.13039/100018503Infraredx, 10.13039/100004339Sanofi, and 10.13039/100009857Regeneron and consulting/speaker fees from Abbott, Amgen, Boston Scientific, Biotronik, Gentuity, Novo Nordic, Medtronic, and Occlutech. Dr Gräni has received funding from the 10.13039/501100001711Swiss National Science Foundation (grant number 200871), InnoSuisse, the Center for Artificial Intelligence in Medicine University Bern, the GAMBIT Foundation, the Novartis Foundation for Medical-Biological Research, and the Swiss Heart Foundation, outside of the submitted work. Dr All other authors have reported that they have no relationships relevant to the contents of this paper to disclose.

## Appendix

Video 1 Intravascular Ultrasound Pullback Performed During Rest The pullback begins in the distal extramural segment, where the lumen is circular. The catheter is then withdrawn through the intramural course, which is identified by progressive ellipticity of the vessel lumen. In these images, the aortic side corresponds to the top of the display and the pulmonary side to the bottom. The pullback continues until only the catheter shaft is visible.

Video 2 Intravascular Ultrasound Pullback Performed During Dobutamine-Atropine Volume Challenge The pullback is initiated in the extramural segment, where the lumen appears circular, and advanced through the intramural course. In this video, the aortic side is at the bottom of the image and the pulmonary side at the top. The pullback continues until only the aortic wall is visualized during both systole and diastole.

Video 3 Demonstration of Rest Pulsatile Lumen Deformation (Change From Diastole to Systole During Rest) in a Pseudo-3D Model, Where All Frames Are Aligned Toward Each Other but Then Displayed on a Straight Line Instead of the Patient-Specific Centerline The regions with the relative highest change from diastole to systole are shown in red.

Video 4 Demonstration of Stress Pulsatile Lumen Deformation (Change From Diastole to Systole During Dobutamine-Atropine Volume Challenge) in a Pseudo-3D Model, Where All Frames Are Aligned Toward Each Other but Then Displayed on a Straight Line Instead of the Patient-Specific Centerline The regions with the relative highest change from diastole to systole are shown in red.

Video 5 Demonstration of Stress-Induced Diastolic Lumen Deformation (Change From Diastole Rest to Diastole During Dobutamine-Atropine Volume Challenge) in a Pseudo-3D Model, Where All Frames Are Aligned Toward Each Other but Then Displayed on a Straight Line Instead of the Patient-Specific Centerline The regions with the relative highest change from rest to stress are shown in red.

Video 6 Demonstration of Stress-Induced Systolic Lumen Deformation (Change From Systole Rest to Systole During Dobutamine-Atropine Volume Challenge) in a Pseudo-3D Model, Where All Frames Are Aligned Toward Each Other but Then Displayed on a Straight Line Instead of the Patient-Specific Centerline The regions with the relative highest change from rest to stress are shown in red.
